# Evaluation of newborn screening in the state of Mato Grosso from 2005 to 2019

**DOI:** 10.1590/1984-0462/2024/42/2022161

**Published:** 2023-08-25

**Authors:** Roseli Divino Costa, Maria de Fátima de Carvalho Ferreira, Thaís de Almeida Rocha, Marcial Francis Galera

**Affiliations:** a Universidade Federal do Mato Grosso, Cuiabá, MT, Brazil.; b Hospital Universitário Júlio Müller, Cuiabá, MT, Brazil.

**Keywords:** National health programs, Program evaluation, Public health, Metabolism, inborn errors, Programas nacionais de saúde, Avaliação de programa, Saúde pública, Erros inatos do metabolismo

## Abstract

**Objective:**

To evaluate quality indicators of the Neonatal Screening Referral Service of the state of Mato Grosso (NSRS-MT) from 2005 to 2019.

**Methods:**

Cross-sectional, retrospective, exploratory, descriptive, and observational study from 2005 to 2019. The following parameters were analyzed: age of newborns at the first collection, time between sample collection and arrival at the laboratory, time between the arrival and release of results and time between requesting the second sample and arrival at the NSRS. The population coverage of the program and the incidence of each clinical situation screened were also analyzed.

**Results:**

NSRS-MT coverage was analyzed and recorded as 76%. The incidence was analyzed for congenital hypothyroidism (CH) 1:1867, phenylketonuria (PKU) 1:33,311, sickle cell disease (SCD) 1:2004, cystic fibrosis (CF) 1:12,663, congenital adrenal hyperplasia (CAH) 1:15,843, and biotinidase deficiency (DB) 1:25,349. The median age (days) at the first consultation was: 44 for HC, 22 for PKU, 60 for DF, 52 for FC, 79 for HAC and 79 for DB. The mean time between exam collection and delivery to the NSRS was 8.4 days; between the arrival and release of results, 9 days; and for the return of recalls, 59 days.

**Conclusions:**

Regarding the coverage of the target population and collection at the ideal age, the NSRS-MT presents values below the national average. However, regarding the mean age at the time of the first consultation, the state's performance is better than the national.

## RESUMO

**Objetivo:**

Avaliar indicadores de qualidade do Serviço de Referência em Triagem Neonatal do Estado de Mato Grosso (SRTN/MT) no período de 2005 a 2019.

**Métodos:**

Estudo transversal, retrospectivo, exploratório, descritivo e observacional, que utilizou dados do formulário FormSUS nos anos de 2005 a 2019. Foram analisados os seguintes parâmetros: idade dos recém-nascidos na primeira coleta, tempo entre coleta da amostra e chegada ao laboratório, tempo entre a chegada e a liberação dos resultados e tempo entre a solicitação da segunda amostra até a chegada ao SRTN. Foram analisadas, também, a cobertura populacional do programa e a incidência de cada situação clínica triada.

**Resultados:**

Cobertura do SRTN-MT: 76%. Incidências: hipotireoidismo congênito (HC) 1:1.867, fenilcetonúria (PKU) 1:33.311, doença falciforme (DF) 1:2.004, fibrose cística (FC) 1:12.663, hiperplasia adrenal congênita (HAC) 1:15.843 e deficiência de biotinidase (DB) 1:25.349. A mediana da idade (dias) na primeira consulta foi: 44 para HC, 22 para PKU, 60 para DF, 52 para FC, 79 para HAC e 79 para DB. A média entre a coleta do exame e a entrega no SRTN foi de 8,4 dias; entre a chegada e liberação dos resultados, de 9 dias; e para o retorno de reconvocados, de 59 dias.

**Conclusões:**

Com relação à cobertura da população alvo e a coleta na idade ideal, o SRTN apresenta valores abaixo da média nacional. Contudo, quanto à idade média no momento da primeira consulta, o desempenho de MT é melhor que a média nacional.

## INTRODUCTION

Neonatal screening (NS) in Brazil was instituted as a program in 2001 when Ordinance No. 822 of the Minister's Office/Ministry of Health (GM/MS)^1^ was published, creating the National Neonatal Screening Program (NNSP). The program aims not only at performing biological screening, but also at ensuring that every child with a confirmed diagnosis receives treatment and a multidisciplinary follow-up through the Neonatal Screening Reference Services (NSRS) in each one of the country's states.^2^ The State of Mato Grosso was qualified in 2001 and, in October of 2002, the Hospital Universitário Júlio Müller (HUJM) was registered as a NSRS.^3^

Successful neonatal screening requires a system that encompasses policy and public funds, high sensitivity and specificity tests, and the follow-up of positive patients by the multidisciplinary team. It is essential that laboratories have quality control programs, guidelines, protocols, standards, continuing education at the collection stations to produce health gains. The quality of collected samples and the timeliness of collection and transport to the laboratory affect the quality and delivery of results.^4^

The Brazilian MS establishes the following neonatal screening indicators: coverage of the NNSP, percentage of collection of the heel prick test on the ideal date, age of the newborn on the date of the first consultation and heel prick test collection locations.^5^ Some quality indicators used in the United States are also used in Brazil, such as time from birth to sample collection, time from sample collection to arrival at the laboratory and time from sample arrival at the laboratory to the release of the results.^6^ In Europe, improvements in neonatal screening were observed from 2010 to 2020, with the use of more advanced methodologies and an increase in the number of screened diseases. This advance occurred due to the joint forces of governments and scientific and patient societies. Performance indicators must be constantly evaluated so as to suggest practices to be adopted.^7^

The NNSP is of great national importance, and it is considered a success within the Unified Health System (SUS). It is being implemented in all Brazilian states and in the Federal District. It privileges the principles of universality, equity, integrality, preservation of autonomy and health care equality. Affected individuals are monitored by a multidisciplinary team in specialized services, aiming at integral healthcare, reducing morbidity and mortality and improving quality of life.^8^ The objective of the present study was to evaluate the quality indicators of the NSRS in the state of Mato Grosso (NSRS-MT) from 2005 to 2019.

## METHOD

A cross-sectional study was carried out using secondary data from the NSRS of Mato Grosso, located at the HUJM of the Federal University of Mato Grosso/ Empresa Brasileira de Serviços Hospitalares (UFMT/EBSERH). The retrospective, descriptive and observational study was based on forms of the Unified Health System (FormSUS) related to the NNSP, sent annually to the MS. The research was authorized by the ethics committee of the HUJM under Certificate of Presentation for Ethical Consideration (CAAE) number 76629317.0.00005541. Data were collected from January 1^st^ 2005 to December 31^st^ 2019. FormSUS is a service of the IT department of SUS (DATASUS) for creating forms on the web, intended to be used by SUS and public partner agencies for activities of public interest.

The variables used in this work were:

- Program coverage in the state of Mato Grosso — the coverage indicator refers to the percentage of newborns who underwent neonatal screening tests in the 1^st^ sample in relation to the number of live births reported in the data source specified for the calculation, in a given geographic space, in the year/period considered.^9^
- Newborns’ age at first collection.
- Average time between collection and sample arrival at the NSRS.
- Average time between sample arrival at the NSRS and results release.
- Average return time of recalls.
- Newborns’ age at first consultation.
- Incidence of diseases screened by the NSRS from 2005 to 2019.
- Number of collection stations registered in the (NSRS-MT).

Descriptive statistics were used to analyze the data. The data were reported as frequency or mean and standard deviations or median and variation. Population coverage was calculated according to the number of neonates screened and the number of births during the study period (newborns tested/live births x 100). To calculate the incidence, the total number of tests performed during the period was used, divided by the total number of cases found.

## RESULTS

**Figure 1:**
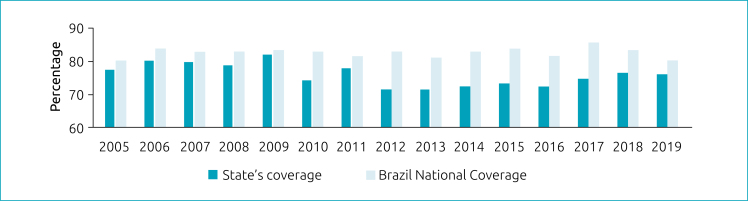
Coverage of neonatal screening in Brazil and in the state of Mato Grosso from 2005 to 2019.

**Figure 2:**
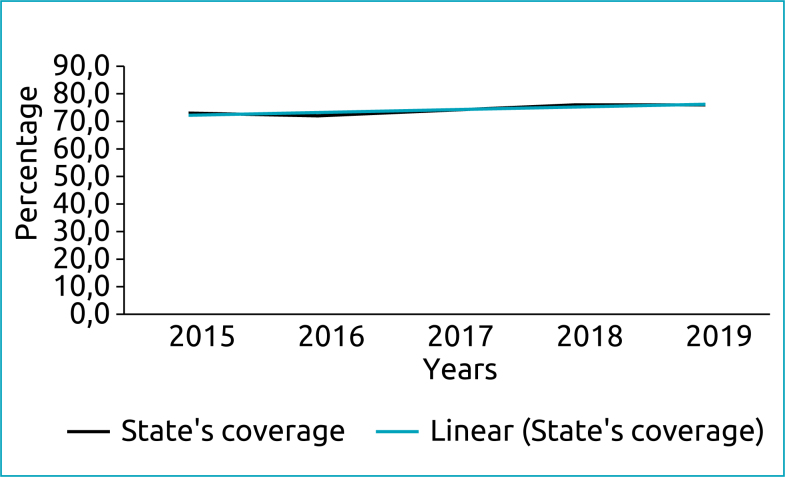
Coverage of neonatal screening in the state of Mato Grosso from 2012 to 2019 showing trend line.

From 2005 to 2019, 599,534 children were screened in the NSRS-MT, out of a total of 792,213 live births. Neonatal screening coverage in the 141 municipalities in the period was 76%, as shown in Figures 1 and 2.

Newborns’ age at collection until the year 2010 was defined as up to the seventh day of life. Aiming at improving indicators and diagnosing other diseases, new age groups were included as of 2011. As of 2011, collection between three and five days of life was considered convenient. From 2005 to 2010, the percentage of collections performed until the seventh day of life was 28%, from eight to 29 days of life 47%, and above 30 days 5%; between 2011 and 2019, the average collection up to five days of life was 30.1%. Most collections were carried out in the age group between six and 30 days (65.9%), and only 4% of the collections were done after 30 days of life, as shown in Table 1.

**Table 1:** Distribution of sample collection according to age in days. Neonatal Screening Referral Service of the state of Mato Grosso from 2005 to 2019.

	Age (days) until the collection of samples					
	0 to 7		8 to 30		>30	
2005	29.2		63.6		7.2	
2006	31.6		62.9		5.5	
2007	35.9		59.5		4.6	
2008	36.8		58.9		4.3	
2009	39.6		56.1		4.3	
2010	37.7		57.6		4.8	
	0 to 2	3 to 5	6 to 8	9 to 14	15 to 30	>30
2011	0.8	26.0	28.7	25.2	14.6	4.7
2012	0.7	24.7	28.9	26.2	14.6	4.9
2013	0.6	29.3	27.5	24.2	14.0	4.4
2014	0.7	30.1	27.8	24.4	13.4	3.6
2015	0.9	29.8	28.4	24.8	12.7	3.4
2016	0.9	29.4	29.9	24.2	12.4	3.2
2017	0.9	35.5	28.9	21.4	10.4	2.9
2018	0.9	29.2	29.7	23.0	11.7	5.5
2019	0.9	28.9	32.2	22.7	11.2	4.1

The average time between collection and delivery of the sample from the collection point to the NSRS was 8.4 days. It should be noted that an increase in this parameter was observed in the last three years analyzed, after a relative stability of about five days on average. The mean time to release results from the NSRS was 10.8 days. However, the values for the years 2017 and 2019 are notably well above the average time observed in the period studied. The average return of recalled samples (altered and inappropriate) was 58.5 days. All data are detailed in Table 2.

**Table 2:** Quality indicators of the Neonatal Screening Referral Service of Mato Grosso, from 2011 to 2019, in relation to the time of collection and arrival of samples at the screening service, time between the arrival of samples at the service and release of results and return of recalled samples.

Years	Days between sample collection and arrival at the NSRS-MT	Days between sample arrival at NSRS-MT and result release	Time between 2nd sample request and arrival at NSRS-MT (days)
2005	12.5	14.8	72
2006	21	9	30
2007	10.3	5.6	16.2
2008	10	5	24
2009	10.8	7.6	37
2010	8	11	78
2011	8.5	8.5	104
2012	7	9	97
2013	5.2	8.2	86
2014	5.2	8.6	73.5
2015	5.1	9.1	58.1
2016	5.1	10.8	65.1
2017	5.5	25.8	64.8
2018	5.8	9.4	36.7
2019	6.6	19.1	35.9
Mean ± SD (median)	8.4 ± 4.1 (7)	10.8 ± 5.2 (9)	58.5 ± 26.4 (64.8)

NSRS-MT: Neonatal Screening Referral Service of Mato Grosso; SD: standard deviation.

Newborns’ age at the first consultation varied greatly according to the disease being screened. In the case of PKU the median was 22 days; on the other hand, for DB it was 78.8 days. Table 3 shows these data in detail.

**Table 3:** Age (in days) at first appointment (median and mean), number of cases and incidence by disease screened by the Neonatal Screening Referral Service of Mato Grosso from 2005 to 2019.

Screened disease	Age at 1^st^ appointment	Age at 1^st^ appointment, Brazil//	Number of diagnosed cases	Incidence
Congenital hypothyroidism*	44	35	321	1:1867
Phenylketonuria*	22	30	18	1:33,307
Sickle cell diseases and other hemoglobinopathies†	60	54	216	1:2775
Cystic fibrosis‡	52	47	22	1:27,251
Congenital adrenal hyperplasia§	61	29	16	1:37,470
Biotinidase deficiency§	79.8	47	10	1:59,953

Age is expressed as median days.

* Data from 2005 to 2019;

† Data from 2009 to 2019;

‡ Data from 2013 to 2019;

§ Data from 2014 to 2019;

// Data from 2016 to 2020.

The most frequent disease detected by the NSRS-MT was HC (1:1,867) and the least frequent was DB (1:59,533). The different incidences of the six diseases screened by the program are described in Table 3.

Sickle cell diseases and other hemoglobinopathies were diagnosed in 216 cases, as follows: FS in 129 cases (incidence of 1:4,337), FSC in 70 cases (incidence of 1:8,564), FC in 15 cases (incidence of 1:39,968) and FSD in two cases (incidence of 1:299,767). As for heterozygotes, the incidence was 1:30.64, with FAS: 1:40.02, FAC 1:131.6 and FAD: 1:20,147.

Up to 2017, there were approximately 330 NS collection stations in the primary care network; in 2019, 672 sample collection stations were recorded in the National Neonatal Screening System database (SISNEO).

## DISCUSSION

NNSP coverage in Brazil from 2016 to 2019 was between 80 and 84%.^9^ The HUJM/UFMT/EBSERH has been performing neonatal screening tests for almost 20 years. The program's coverage in the 2016–2019 period was below the national coverage with a descending trend line. However, when analyzing the coverage in the last eight years of the study, there is a clear rising trend, denoting recovery.

Several factors contribute to this result: socioeconomic and cultural problems (families that do not prioritize the program or give it importance); the extensive territory of Mato Grosso and its peculiarities, such as distant collection points, a precarious road network and its specific climatic conditions. Systemic/structural problems are also highlighted, as there is no formal program to encourage collection, not all health units are qualified for collection, opening hours are restricted, the team of professionals involved in the program are poorly trained, and they fail in sensitizing the target population.^10^ Due to the laboratory methodology employed, the collection of biological samples in the state is carried out in Basic Health Units and not in maternity hospitals. This occurs because the technique used to detect phenylalanine levels requires the newborn to have been fully breastfed for at least 48 hours of life. In 2019, DATASUS^11^ presented 959 health teams registered in primary care in the state of Mato Grosso. Of these, only 70% were registered in the Neonatal Screening Information System (NSIS) as collection sites, which may also be one of the reasons for the low screening coverage in the state. Until 2017, there were approximately 330 NS collection sites in the basic health network; in 2019, 672 posts were registered in the NSIS database. The supposed expansion of the collecting network did not actually occur. With the change of the old neonatal screening information system to NSIS, it was possible to register all the units that already collected data and had not been included. That is, there must have been an increase, but not as great as the data show, and it did not cause a significant impact on the coverage of the NSRS-MT.

The MS recommends that collection be performed from the third to the fifth day after birth. The state of Mato Grosso was below the national average: only 54.8% of collections were done as recommended by the MS.^8^ Timely collection allows the identification of diseases in the pre-symptomatic phase and early interventions.^12^ Other factors also lead to decreased program efficiency: time to deliver samples to the laboratory and time to release results to the health facilities. Most collections were performed between the sixth and 30^th^ day after birth. Changes in these parameters need the involvement of municipal and state managers, better organization and operation of the collection network, as well as the establishment of educational campaigns involving health professionals and the population.^13^ It is recommended that biological samples do not remain in the collection units for more than two days.^8^ There is no data, however, about the ideal period for their arrival at the NSRS. These parameters were worse in the last three years analyzed in the study. It is possible to relate this performance to the lack of adequate, established routines, the need for continued education that highlights the program's importance and the lack of audit mechanisms by the state's Health Department.

It is recommended that results be delivered within seven days.^8^ The state of Mato Grosso presents data compatible with this requirement. However, in 2017 and 2019, the period was greater that the recommended one. This fact must be related to the difficulty in acquiring laboratory supplies during those years.

The incidence of PKU in a study carried out previously in the state of Mato Grosso (1:33,068) was similar to the data obtained in this research,^14^ as well as in Santa Catarina and Tocantins (1:28,000).^15^ The most prevalent disease in newborn screening programs is CH. The incidence has ranged from 1:1,030 to 1:2,679 for live births.^16^ It is believed that the reduction in the cut-off point of the thyroid stimulating hormone (TSH) dosage, the increase in the survival of preterm newborns together with environmental and ethnic factors were associated with an increase in this incidence over the years. The data from the present study show a higher incidence of CH when compared with the 2009 NSRS survey in Ribeirão Preto, which was 1:2,595,^17^ and the data obtained by Silvestrim, Leone and Leone (2010) in a previous study also in the state of Mato Grosso, where the prevalence (1:2234) was evaluated in only one year.^18^ In Brazil, there are different incidences of FD depending on the ethnic differences in the population, but the highest incidence was found in Bahia (1:650). While in the southeastern and northeastern regions incidences ranged from 1:1,300 to 1:4,000, in the southern states, where there is a strong European influence, incidences range from 1:13,500 to 1:11,000. The state of Mato Grosso, because of its highly mixed population, presents intermediate values.^19^ As for heterozygotes, the incidence in the state of Mato Grosso was close to that of São Paulo 1:35 and Minas Gerais 1:30, Bahia 1:17, Rio de Janeiro 1:20, and Goiás 1:25.^20^ The estimated incidence of CF in Brazil is 1:7,576 live births, below that found in the state of Mato Grosso; however, it presents regional differences, with higher values in the southern states.^20^ The highest incidence of CAH was of the salt-wasting form. The incidence of severe forms can range from 1:16,000 to 1:20,000,^21^ similar to that found in the state of Mato Grosso. The incidence of DB varies from 1:40,000 to 1:60,000 births worldwide.^22^ The combined estimated incidence of partial and profound biotinidase deficiency was 1:13,909 live births in Minas Gerais from 2013 to 2018,^23^ above that found in the state of Mato Grosso.

Newborns’ age at the time of the first appointment is relevant for the evaluation of a referral service. The national median for phenylalanine was 30 days, above the median found in the state of Mato Grosso, showing effectiveness and fulfilling the role of the RSNS on the population; unlike children diagnosed with CH, as the national median was 35 days, below that found in the state of Mato Grosso. The median age at the time of the first appointment in the state of Mato Grosso for SCD and other hemoglobinopathies was little above the national average, which is 54. As for CF, newborns’ median age at the first appointment was close to the national value; it should be reminded that early detection allows access to specialized treatment, and is essential to modify the prognosis of the disease. In the case of patients diagnosed with CAH presenting the severe, salt-wasting form, the newborns’ median age on the date of the first appointment is very high in the state, with the national median being 29 days. As for BD, the national median age was 47 days; this indicator needs to be improved by the NSRS-MT, as the number found is much higher.^9^ There are no indicators defining the ideal period for the first appointment. Guidelines only suggest consultation should occur as soon as possible.^6^

Regarding coverage of the target population and collection at the ideal age, the NSRS-MT presents values below the national average. However, with regard to the mean age at the time of the first consultation, the state's performance is better than the national average. The fact that the data source does not allow a more detailed analysis of the information can be cited as a limitation of this study. In addition, the NSIS Program made available by the MS does not have its source code released, thus hampering more elaborate reports.

The NSRS-MT has a reasonable physical structure, a multidisciplinary team both in the clinic and in the laboratory, but, despite these aspects, indicators still leave much to be desired. A joint action of all the spheres involved would have a synergistic effect in improving services and, consequently, indicators. Actions can be understood as the expansion of technical staff, awareness campaigns for the population and the Health Units about the relevance and need for adherence to the program, ongoing training of the collection teams, guaranteed acquisition of laboratory supplies and expansion of their physical space, also aiming at the urgent expansion of the NNSP based on Law No. 14,154 of May 26, 2021.^24^

## Data Availability

The database that originated the article is available with the corresponding author.
